# Permanent Pacemaker Implantation After TAVI and Its Association with Survival: Single-Center Cohort and Nationwide Validation

**DOI:** 10.3390/jcm15062288

**Published:** 2026-03-17

**Authors:** Gudrun Lamm, Cecilia Veraar, Philipp Höbart, Matthias Granner, Maximilian Will, Konstantin Schwarz, Christian Nitsche, Roya A. Mousavi, Johann Auer, Hendrik J. Ankersmit, Matthias Hammerer, Uta C. Hoppe, Julia Mascherbauer

**Affiliations:** 1 Department of Internal Medicine 3, University Hospital St. Pölten-NOE LGA, Karl Landsteiner University, 3100 St. Pölten, Austria; 2 Department of Cardiology, Medical University of Vienna, 1090 Vienna, Austria; 3 Mount Sinai Fuster Heart Hospital, New York, NY 10029, USA; 4 Department of Internal Medicine I with Cardiology and Intensive Care, St. Josef Hospital Braunau, 5280 Braunau am Inn, Austria; 5 Clinic of Thoracic Surgery, Medical University of Vienna, 1090 Vienna, Austria; 6 Laboratory for Cardiac and Thoracic Diagnosis, Regeneration and Applied Immunology, Medical University of Vienna, 1090 Vienna, Austria; 7 Department of Internal Medicine II, Paracelsus Medical University of Salzburg, 5020 Salzburg, Austria

**Keywords:** aortic stenosis, transcatheter aortic valve implantation, pacemaker, mortality

## Abstract

**Background/Objectives:** Permanent pacemaker (PM) implantation is a well-recognized complication of transcatheter aortic valve implantation (TAVI), but its long-term prognostic impact remains uncertain. To evaluate the association between PM implantation and all-cause mortality in TAVI recipients. **Methods:** We performed a post hoc analysis of a prospective single-center TAVI registry (2016–2020). The primary endpoint was all-cause mortality at 1 and 5 years. Cox regression and Kaplan–Meier analyses were applied. Validation was performed using the nationwide AUTHEARTVISIT claims database. **Results:** Among 1114 consecutive TAVI patients (mean age 81  ±  5.8 years; 49.8% female), 120 (10.8%) had a pre-existing PM (Pre-PM), and 153 (13.7%) received a new PM within 30 days post-TAVI (Post-PM). Post-PM patients were older (*p* = 0.006), more often male (*p* < 0.001), had higher Troponin T levels (*p* = 0.002), more pre-existing right bundle branch block (*p* < 0.001), and longer QRS duration (*p* < 0.001) compared to patients without PM. In multivariate analysis, one-year mortality was associated with Troponin T (*p* = 0.002) and NT-proBNP (*p* = 0.002) serum levels. Pre- or Post-PM status was not associated with 1-year mortality (*p* = 0.455, *p* = 975). However, Pre-PM status was independently associated with 5-year mortality (HR 1.4, 95% CI: 1.0–1.9, *p* = 0.03), whereas Post-PM status was not (HR 1.2, 95% CI: 0.8–1.6, *p* = 0.22). Findings were confirmed in the nationwide AUTHEARTVISIT cohort. **Conclusions:** In this large, real-world TAVI cohort with national validation, Post-PM status was not associated with mortality at 1 or 5 years. By contrast, Pre-PM identified patients at higher long-term risk, possibly reflecting underlying cardiac disease.

## 1. Introduction

Transcatheter aortic valve implantation (TAVI) has become an established treatment for patients with severe aortic stenosis across the entire risk spectrum. Initially reserved for inoperable or high-risk patients, TAVI is now frequently performed in intermediate- and low-risk individuals, with a continuously rising number of procedures worldwide [1]. As procedural safety has improved, focus has shifted to long-term outcomes and complications that may impact prognosis.

One of the most common adverse events following TAVI is the development of conduction disturbances, often necessitating permanent pacemaker (PM) implantation. While newer generation devices and improved implantation techniques have reduced the incidence of paravalvular leaks, they have not consistently decreased the need for PM implantation [2,3]. Notably, PM rates vary significantly between different valve platforms, with self-expanding valves showing higher rates than balloon-expandable systems [4,5]. In light of the expanding TAVI population, the potential long-term consequences of pacing—such as electrical dyssynchrony, heart failure, and device-related complications—are gaining clinical and ethical importance [6,7,8].

However, the prognostic significance of post-TAVI PM implantation remains controversial. Some studies and meta-analyses have suggested associations with increased long-term mortality and heart failure hospitalizations [4,9,10,11], while others—including large national registries—have found no negative impact [12,13]. Methodological differences, heterogeneous patient populations, and limited adjustment for confounders complicate interpretation.

In this study, we aimed to evaluate the impact of permanent PM implantation on short- and long-term all-cause mortality after TAVI in a large, unselected single-center cohort, complemented by external validation using a nationwide Austrian dataset [14]. Particular attention was paid to the distinction between pre-existing and procedure-related PMs and to known predictors of pacing in order to contribute clinically relevant evidence to this ongoing debate. To ground our analysis in current evidence, we additionally conducted a structured literature search to synthesize contemporary knowledge on PM implantation after TAVI.

## 2. Methods

### 2.1. Study Design and Population

This is a post hoc analysis of a prospective, single-center observational cohort including all consecutive patients who underwent TAVI at the Department of Cardiology, University Hospital St. Pölten (Austria), between 1 January 2016, and 31 December 2020. The primary objective was to compare 1-year and 5-year all-cause mortality between patients requiring permanent PM implantation within 30 days after TAVI (Post-PM) and those not requiring a PM (No PM). Additionally, we assessed mortality among patients with a pre-existing PM at the time of TAVI (Pre-PM). The study was conducted in accordance with the Declaration of Helsinki (2013 revision) and approved by the local ethics committee of Karl Landsteiner University (EK1066/2022).

### 2.2. Procedural Management and Follow-Up

Patient eligibility for TAVI was determined by a multidisciplinary heart team. Procedures were performed primarily via transfemoral access (99.6%) under conscious sedation. In four cases, subclavian access with general anesthesia was used. Device selection was left to the operator’s discretion. Temporary PMs were placed via femoral venous access in all patients without a prior PM or ICD. These were removed post-procedurally in the absence of an indication for permanent pacing. ECGs were obtained at baseline, immediately post-procedure, and daily for the first three days. All patients underwent continuous cardiac monitoring for ≥72 h and received transthoracic echocardiography prior to discharge.

Permanent PM implantation after TAVI in most cases of high-grade atrioventricular block was performed in accordance with European Society of Cardiology (ESC) guidelines applicable at the time [15,16].

Follow-up visits were conducted at 1 year either on site or via the referring physician. Thereafter, annual telephone interviews were performed to assess vital status and late PM implantations, with follow-up continuing up to 8 years post-procedure. Vital status was cross-verified using data from Statistics Austria.

### 2.3. Data Collection

The institutional TAVI registry collected comprehensive data including demographics (age, sex, height, and weight), medical history, TAVI indication, and 1- and 5-year mortalities. Laboratory, ECG, and echocardiographic parameters were recorded pre-procedurally and postoperatively on day 1. Survival data were censored at 31 December 2023.

### 2.4. External Validation—AUTHEARTVISIT Study

To validate our findings and strengthen their generalizability, we analyzed data from the AUTHEARTVISIT study [14]. This dataset includes anonymized national health insurance data of all patients who underwent TAVI in Austria between 1 January 2010, and 31 December 2020. Patients were stratified based on whether they received a permanent PM within 30 days post-TAVI.

### 2.5. Structured Literature Search

We conducted a structured literature search including both original studies and meta-analyses. Studies were classified by outcome, distinguishing those reporting no significant effect of Post-PM on mortality from those demonstrating a significant effect.

### 2.6. Endpoints

The primary endpoint was all-cause mortality at 1 and 5 years following TAVI.

### 2.7. Statistical Analysis

Continuous variables are expressed as the mean ± standard deviation (SD) and were compared using two-tailed *t*-tests. Univariate and multivariate logistic regression analyses were performed to determine factors associated with PM implantation. Cox proportional hazards models were used to assess associations between variables and 1- and 5-year mortalities. Only variables with *p* < 0.05 in the univariate analysis were included in the multivariate models, except for creatinine and Troponin T, which were excluded due to collinearity with renal function and serum NT-proBNP. Continuous variables were dichotomized at the median for regression analyses.

Survival analyses were performed using the Kaplan–Meier method. Curves were generated for each group, and the number at risk, events, and censored observations were reported annually. Median survival times and yearly survival probabilities with 95% confidence intervals (CIs) were calculated. Group comparisons were made using the log-rank test.

All statistical analyses were conducted using SPSS version 30 (IBM Corp., Armonk, NY, USA), GraphPad Prism 9 (GraphPad Software, La Jolla, CA, USA), and R version 4.4.1.

## 3. Results

**Figure 1:**
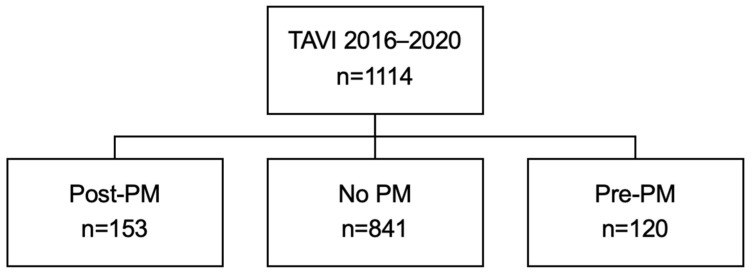
CONSORT statement of patient inclusion. A total of 1114 patients were analyzed, among whom 120 (10.8%) received a PM prior to TAVI and accordingly were excluded from the primary analysis, 841 (75.5%) patients did not require a PM implantation, and 153 (13.7%) underwent PM implantation within 30 days post TAVI.

Between 1 January 2016 and 31 December 2020, 1114 consecutive patients (mean age 81  ±  5.8 years; 555 (49.8%) female) underwent TAVI (927 (83.2%) with balloon-expandable transcatheter heart valves (THVs)) and were followed for a mean of 5.6 ± 1.3 years. Among them, 120 patients (10.8%) already carried a permanent PM at the time of TAVI, including 3 with cardiac resynchronization therapy. Among the remaining 994 patients, 153 (15.4%) underwent new PM implantation within 30 days following TAVI (Figure 1).

Information on the indication for pre-existing cardiac implantable electronic devices was available. Among patients with implantable cardioverter-defibrillators (ICD; *n* = 9), the most frequent indication was ischemic cardiomyopathy (*n* = 5), followed by prior cardiopulmonary resuscitation (*n* = 3) and dilated cardiomyopathy (*n* = 1). Three patients carried a cardiac resynchronization therapy device (CRT), all implanted in the context of ischemic cardiomyopathy (*n* = 3). Among patients with pre-existing pacemakers, the most common indications were advanced atrioventricular block (*n* = 40) and sick sinus syndrome (*n* = 43), whereas the remaining patients received a pacemaker for other indications (*n* = 15).

From the AUTHEARTVISIT national dataset, 6865 patients (mean age 81 ± 5.8 years), 3803 (55.4%) female were analyzed, among whom 776 (11.3%) were Post-PM cases.

### 3.1. Baseline Clinical and Demographic Characteristics

The baseline clinical and procedural characteristics of Pre-PM, Post-PM, and No PM patients are summarized in Table 1. In brief, compared to No PM patients, Post-PM patients were older (81.9 ±  5.5 vs. 80 ±  5.7 years, *p* = 0.006), less likely to be female (71 (46.4%) vs. 445 (52.9%), *p* < 0.001), and had higher rates of peripheral artery disease (16 (10.5%) vs. 53 (6.3%), *p* = 0.019). They presented with higher Troponin T levels (28.3 µg/L (IQR 18–32.1) vs. 23 µg/L (IQR 16–37), *p*= 0.002), higher rates of pre-existing right bundle branch block (RBBB, 43 (28.1%) vs. 27 (3.2%), *p* < 0.001), and a longer QRS duration (110 ms (IQR 90–140) vs. 100 ms (80–110), *p* < 0.001).

Compared to Pre-PM patients, Post-PM patients were more likely to be female (71 (46.4%) vs. 39 (32.5%), *p* < 0.001), had less atrial fibrillation (AF, 55 (35.9%) vs. 64 (53.3%), *p* = 0.002), lower N-terminal pro-brain natriuretic peptide (NT-proBNP) levels (1273 (IQR 468–2779) vs. 2593 (IQR 1081–5055), *p* < 0.001), and presented with higher left ventricular ejection fraction (LVEF, 60% (IQR 60–60) vs. 55% (IQR 35–60), *p* < 0.001). Furthermore, they had more RBBB (43 (28.1%) vs. 3 (2.5%), *p* < 0.001), left bundle branch block (LBBB, 11 (7.2%) vs. 2 (1.7%), *p* = 0.007), left anterior hemiblock (LAH, 52 (36.1%) vs. 23 (20%), *p* < 0.001), and shorter QRS duration (110 ms (IQR 90–140) vs. 130 ms (IQR 110–150), *p* < 0.001).

The demographic characteristics of the AUTHEARTVISIT cohort are presented in Table 2. In brief, No PM patients were more likely to be female (3400 (55.8%) vs. 403 (51.9%), *p* = 0.043) and had less hyperlipidemia (1494 (24.5%) vs. 218 (28.0%), *p* = 0.035).

### 3.2. Procedural Data

Table 1 shows the procedural characteristics. The majority of patients (83.2%) received balloon-expandable THVs. Compared to Pre-PM patients, Post-PM recipients were implanted with smaller aortic bioprostheses (26.9 mm ±  2.2 vs. 27.2 mm ± 2.0, *p* < 0.001). In 99.6%, a femoral access was used.

### 3.3. Pacemaker Implantation After TAVI

All patients in need of a permanent PM who were in sinus rhythm received dual-chamber pacing, and all patients in AF received single-chamber PMs. Among Post-PM patients, 35.9% underwent VVI PM implantation. Neither CRT devices nor conduction system-pacing nor devices with ICD function were implanted.

### 3.4. Predictors of PM Implantation Within 30 Days of TAVI

Predictors of Post-PM are demonstrated in Table 3. In univariate logistic regression, the following variables were significantly associated with PM implantation within 30 days: age (*p* = 0.040), RBBB (*p* < 0.001), LAH (*p* < 0.001), LVEF (*p* = 0.020), and prosthesis size (*p* = 0.002). In multivariate analysis, only RBBB (*p* < 0.001), LAH (*p* = 0.020), LVEF (*p* = 0.007), and prosthesis size > 26 mm (*p* = 0.002) remained independently associated with the need for PM implantation after 30 days, while valve type (83.2% balloon-expandable) was not.

### 3.5. Outcome at One and Five Years

**Figure 2:**
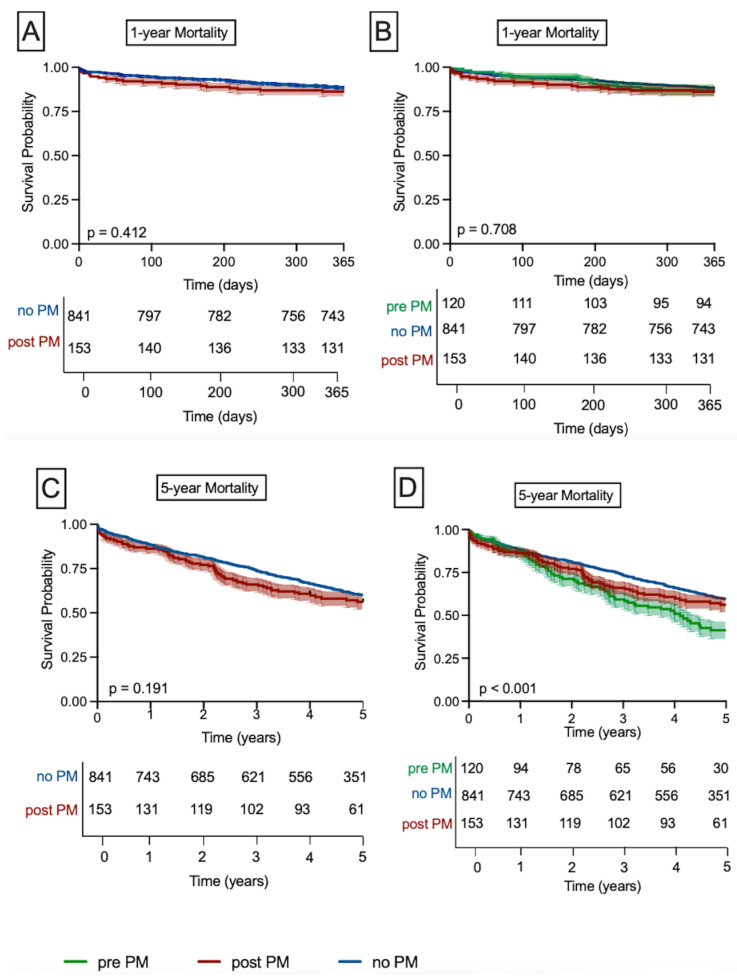
One- and five-year mortalities among patients requiring PM insertion. There was no difference in 1-year survival probability among Post-PM patients compared to No PM patients, as depicted in (**A**). In addition, there was no difference in 1-year survival probability among Pre-PM, Post-PM, or No PM patients (**B**). Further, there was no difference in 5-year mortality among No PM patients compared to Post-PM patients, as shown in (**C**). By contrast, there was a significant difference in 5-year survival probability among Pre-PM patients compared to Post- or No PM patients (**D**).

Kaplan–Meier survival estimates (Figure 2A) demonstrated no significant difference in 1-year all-cause mortality between patients with and without PM (log-rank *p* = 0.41). Likewise, stratification by pacemaker status (No PM, Pre-PM, and Post-PM) showed comparable 1-year survival rates (Figure 2B; *p* = 0.71). At 5 years, mortality was comparable between the Post-PM and No PM groups (Figure 2C; *p* = 0.191), whereas patients with any PM exhibited higher mortality than those without, largely attributable to excess deaths in the Pre-PM group (Figure 2D, *p* < 0.001).

In univariate Cox regression for 1-year mortality, neither Post-PM (HR 1.10, 95% CI 0.70–1.90, *p* = 0.455) nor Pre-PM (HR 0.90, 95% CI 0.50–1.70, *p* = 0.975) status was associated with early mortality. By contrast, several baseline parameters demonstrated significant associations: Troponin T > 24 pg/mL (HR 3.3, 95% CI 2.1–5.3, *p* < 0.001), NT-proBNP > 1532 ng/L (HR 2.8, 95% CI 1.8–4.4, *p* < 0.001), LVEF < 60% (HR 1.7, 95% CI 1.2–2.4, *p* < 0.001), EuroSCORE II ≥ 5 points (HR 1.7, 95% CI 1.2–2.4, *p* = 0.002), and chronic kidney disease (CKD, HR 2.6, 95% CI 1.4–4.9, *p* = 0.002). After multivariate adjustment, only Troponin T (HR 2.2, 95% CI 1.3–3.8, *p* = 0.002) and NT-proBNP (HR 2.2, 95% CI 1.3–3.8, *p* = 0.002) remained independently associated with 1-year mortality, as detailed in Table 4.

At the 5-year follow-up, Pre-PM status was significantly associated with increased all-cause mortality (HR 1.6, 95% CI 1.2–2.1, *p* < 0.001), whereas Post-PM status did not reach statistical significance (HR 1.2, 95% CI 0.9–1.5, *p* = 0.181). Other univariate predictors of adverse long-term outcome included age > 82 years (HR 1.3, 95% CI 1.1–1.6, *p* < 0.001), AF (HR 1.5, 95% CI 1.3–1.9, *p* < 0.001), CKD (HR 2.2, 95% CI 1.4–3.2, *p* < 0.001), Troponin T (HR 2.0, 95% CI 1.6–2.4, *p* < 0.001), and NT-proBNP (HR 1.8, 95% CI 1.5–2.3, *p* < 0.001). In the multivariate model, Pre-PM status remained an independent predictor of long-term mortality (HR 1.4, 95% CI 1.0–1.9, *p* = 0.030), while Post-PM status remained non-significant (HR 1.2, 95% CI 0.8–1.6, *p* = 0.220). Independent covariates associated with late mortality comprised age > 82 years (HR 1.3, 95% CI 1.1–1.7, *p* = 0.004), AF (HR 1.3, 95% CI 1.0–1.7, *p* = 0.006), CKD (HR 2.0, 95% CI 1.1–3.8, *p* = 0.020), Troponin T (HR 1.6, 95% CI 1.2–2.0, *p* < 0.001), and NT-proBNP (HR 1.4, 95% CI 1.1–1.8, *p* = 0.006), as depicted in Table 5.

### 3.6. AUTHEARTVISIT National Dataset

**Figure 3:**
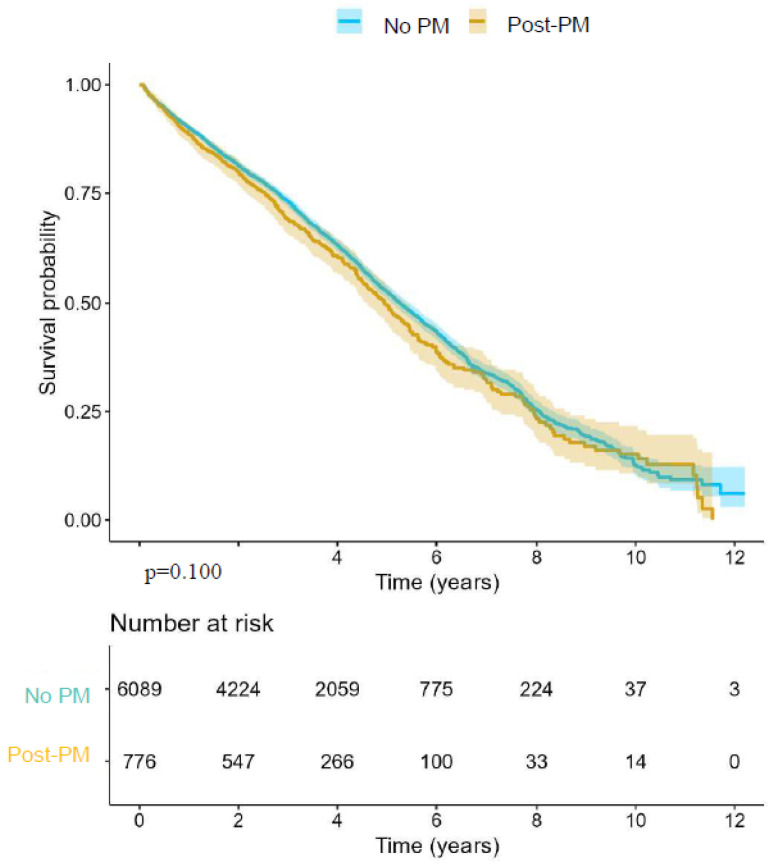
Long-term mortality of 6865 Post- and No PM patients. Data from the AUTHEARTVISIT study. There was no difference in 12-year survival probability among Post-PM patients compared to No PM patients.

Kaplan–Meier survival curves from the AUTHEARTVISIT cohort are shown in Figure 3, comparing Post-PM and No PM patients. The median follow-up time was 4.25 years. There was no statistically significant difference in all-cause mortality between the two groups (log-rank *p* = 0.100). The estimated median survival was 4.95 years (95% CI: 4.60–5.35) for Post-PM patients and 5.26 years (95% CI: 5.11–5.46) for No PM patients (HR 1.06; 95% CI: 0.98–1.11, *p* = 0.1).

### 3.7. Overview of Key Studies on Post-TAVI Permanent PM and Clinical Outcomes

A literature overview is depicted in Table 6. Across the included studies, rates of Post-PM ranged between 12% and 31%. Follow-up durations extended up to 10 years. Several registries and cohort studies, including SWEDEHEART [12], UK-TAVI [17], PARTNER 2 S3 [18], TAVI-NOR [19], Hochstadt et al. [13], and the present study, reported no significant association between Post-PM implantation and long-term all-cause mortality. On the other hand, SwissTAVI [9], FRANCE-TAVI [20], and the Danish cohort [10] identified an increased risk of mortality among Post-PM patients.

## 4. Discussion

In this large, single-center, all-comer TAVI cohort, PM implantation within 30 days was not associated with an increased risk of mortality at 1 or 5 years, whereas pre-existing PM identified patients at greater long-term risk. We validated the finding of a neutral impact of Post-PM using the nationwide AUTHEARTVISIT dataset comprising over 6800 TAVI recipients [14].

Although the need for post-TAVI PM is an established procedural complication, its impact on short- and long-term outcomes remains debated. Evidence is limited, with only a handful of dedicated studies [9,10,13,15] and two recent meta-analyses [4,11], and results remain inconsistent. A summary of these data is provided in Table 6.

The findings of the present study align with the population-based SWEDEHEART registry [12], which included 3420 transfemoral TAVI patients and reported no significant differences in mortality, heart failure hospitalization, or endocarditis between patients with and without Post-PM. Similarly, Hochstadt et al. [13] found no association between Post-PM status and long-term mortality—even among patients with high pacing burden—whereas Pre-PM status was linked to significantly worse outcomes (HR ≈ 1.53; *p* = 0.002). Moreover, declines in LVEF occurred more frequently among Post-PM patients.

By contrast, the SwissTAVI registry [9] recently reported a significant increase in long-term mortality among Post-PM patients (*n* = 13,360, TAVI 2011–2022). Several differences between this registry and the aforementioned studies warrant attention. Balloon-expandable valves were used in <50% of SwissTAVI patients compared to ~80% in SWEDEHEART patients and our cohort, and Post-PM rates differed substantially (SwissTAVI 20%, SWEDEHEART 14%, present study 18%). Furthermore, the Kaplan–Meier curves for the SwissTAVI cohort crossed repeatedly, suggesting possible non-proportional hazards, yet no time-stratified or landmark analyses were performed. Although *p*-values reached statistical significance, absolute survival differences remained small. Similarly, a Danish single-center study [10] of >800 patients (79% self-expanding valves) reported increased late mortality, heart failure hospitalizations, and reduced LVEF among Post-PM patients.

When contextualizing our findings within prior evidence, results across individual studies have been heterogeneous [10,12,13]. Cohorts with a high proportion of balloon-expandable valves—such as ours, SWEDEHEART [12], Hochstadt et al. [13], and PARTNER 2 S3 [18]—consistently found no significant association between Post-PM and long-term mortality. By contrast, studies with greater use of self-expanding valves, including SwissTAVI [9] and the Danish study [10], reported an increased mortality risk. These discrepancies may reflect differences in valve technology, implantation techniques, and unmeasured confounders. Notably, in the smaller TAVI-NOR [19] cohort, which reported the highest Post-PM rate and frequent use of self-expanding and mechanically expandable valves, no difference in long-term mortality was observed—a paradox that is potentially attributable to liberal implantation practices and limited statistical power. In our study, the proportion of self-expanding valves was very low, which precludes clinically meaningful subgroup analyses.

Two recent meta-analyses further illustrate this complexity. Faroux et al. [4] pooled data from >50,000 patients across 30 studies and reported increased risks of all-cause mortality (RR = 1.17, 95% CI 1.11–1.25) and heart failure hospitalization (RR = 1.18, 95% CI 1.03–1.36) among Post-PM patients, although cardiac death was not significantly increased. In line with this, Zito et al. [11] performed a systematic review of 31 studies (*n* = 51,069) and found similar results: increased all-cause mortality (RR = 1.18, 95% CI 1.10–1.25) and higher heart failure rehospitalizations (RR = 1.32), but no difference in stroke or myocardial infarction. Both meta-analyses are limited by retrospective designs, heterogeneous methodologies, and incomplete adjustment for valve type or pacing burden, highlighting the need for more granular, contemporary data.

Mechanistically, chronic right ventricular pacing can cause dyssynchrony and adverse remodeling, especially at high pacing burdens [21,22], but these effects usually require years to manifest. This partly explains why Pre-PM status was associated with worse outcomes in our cohort. Additionally, the Pre-PM group had baseline characteristics indicating a higher comorbidity burden that might also have contributed to the higher rate of adverse events and increased mortality in this cohort.

Post-TAVI conduction disturbances, on the other hand, are often transient and may resolve spontaneously, especially with modern high-implantation techniques [23]. Leadless pacemakers [24] may further reduce pacing-related complications, but long-term data remain limited. Finally, given the advanced age of our study population, pacing may contribute only marginally to overall mortality, where competing comorbidities dominate.

### Strenghts and Limitations

To enhance statistical power and generalizability, we combined our prospective single-center cohort with the large nationwide AUTHEARTVISIT dataset [14], which strengthened external validity. Further strengths of our study include a large sample size, standardized procedural protocols, guideline-based PM indications, long follow-up, and independent validation across two datasets.

However, several limitations should be acknowledged. First, claims-based data are inherently limited by potential miscoding, lack of device-level details, and reduced clinical granularity. Second, pacing-specific parameters—such as pacing burden, PM dependency, and use of conduction-system pacing or leadless devices—were unavailable, which restricts mechanistic interpretation and precludes assessment of the potential impact of chronic right ventricular pacing on long-term outcomes. Moreover, no systematic information was available regarding recovery of conduction disturbances and long-term pacemaker dependency. Additionally, we had no information regarding device-related parameters such as lead position and therefore could not incorporate such data into the analyses. Although ventricular pacing minimization algorithms were routinely activated, we had no access to device programming details. Third, detailed information on the timing of pre-existing PM implantation was not available in the majority of patients, as most were referred from external centers. This limits mechanistic interpretation of the association between Pre-PM status and long-term mortality. Fourth, procedural factors such as CT-based implantation depth and valve positioning were not analyzed. Fifth, outcomes beyond all-cause mortality—including cause-specific mortality, heart failure hospitalizations, and endocarditis rates—were not captured. The lack of information on cause-specific mortality limits interpretation regarding cardiovascular versus non-cardiovascular death. Finally, valve selection was left to the operator’s discretion. The presence of bulky LVOT calcification could have triggered implantation of a self-expandable valve in some patients. However, our team is usually more comfortable implanting balloon-expandable valves in patients with high-risk features, resulting in a much higher rate of balloon-expandable devices in the overall cohort. This reflects clinical practice at our institution but may limit the generalizability of the results to other cohorts with a higher rate of self-expandable valves. Additionally, no subgroup analysis by TAVI platform could be performed, which limits direct comparison with studies reporting divergent outcomes.

## 5. Conclusions

Taken together, our findings indicate that in contemporary, balloon-expandable–dominated elderly populations, post-TAVI PM implantation does not impose a clinically relevant survival disadvantage. By contrast, PM implantation before TAVI was associated with significantly worse long-term outcomes, likely reflecting underlying conduction disease and comorbid burden.

However, recent registry data and meta-analyses suggest that increased mortality risk may exist in subgroups treated predominantly with self-expanding valves. As TAVI expands to younger, lower-risk populations, careful evaluation of the long-term impact of pacing-related remodeling and conduction disturbances becomes increasingly important. Future research should focus on subgroup analyses by valve type, pacing burden quantification, and the role of novel technologies, such as conduction-system pacing and leadless pacemakers, to optimize device selection and patient outcomes. In younger patients with decades of pacing exposure ahead, the potential for long-term harm must be carefully balanced against procedural safety, device costs, and healthcare resource allocation.

### Clinical Perspective

**What is known:** The impact of pacemaker (PM) implantation on outcomes after transcatheter aortic valve implantation (TAVI) remains controversial.

**What is new:** This large, real-world TAVI cohort with national validation demonstrated no increased mortality at 1 or 5 years after implantation of a new PM within 30 days after TAVI. However, a pre-existing PM prior to TAVI identified patients at increased risk of long-term mortality, possibly reflecting underlying cardiac disease.

**What is next:** This cohort mainly comprised patients in their eighties at increased risk, therefore, the findings may not be generalizable to younger or low-risk populations. Further studies in younger patients are needed to clarify the long-term impact of TAVI-associated PM implantation.

## Figures and Tables

**Table 1:** Baseline clinical and demographic data.

	No PM *n* = 841	Post-PM *n* = 153	Pre-PM N = 120	*p*-Value
**Demographic data**				
Age (years) ^##^	80 ± 5.7	81.9 ± 5.5	82.5 ± 5.8	**0.006**
Gender (female) ^#^	445 (52.9)	71 (46.4)	39 (32.5)	**<0.001**
BMI (kg/m^2^) ^##^	28.0 ± 7.5	28.5 ± 12.7	28.5 ± 10.7	0.246
**Comorbidities and biomarkers:**				
DM ^#^	282 (33.5)	40 (33.3)	43 (28.1)	0.417
CAD ^#^	420 (49.9)	71 (46.4)	67 (55.8)	0.298
PAD ^#^	53 (6.3)	16 (10.5)	15 (12.5)	**0.019**
AF ^#^	311 (37.0)	55 (35.9)	64 (53.3)	**0.002**
Euroscore II ^##^	4.8 ± 5.5	5.4 ± 5.9	5.9 ± 5.3	0.246
Troponin T (µg/L) *	23 (16.0; 37.0)	28.3 (18.0; 32.1)	30.4 (20.1; 41.0)	**0.002**
NT-proBNP (ng/L) *	1508 (553; 3755)	1273 (468; 2779)	2593 (1081; 5055)	**<0.001**
CRP (mg/dL) *	0.3 (0.1; 0.7)	0.2 (0.1; 0.4)	0.3 (0.1; 0.8)	0.208
Creatinine (mg/dL) *	1.0 (0.9; 1.3)	1.1 (0.9; 1.4)	1.1 (0.9; 1.4)	**0.003**
**ECG-parameters (prior to intervention):**				
RBBB ^#^	27 (3.2)	43(28.1)	3 (2.5)	**<0.001**
LBBB ^#^	85 (10.1)	11 (7.2)	2 (1.7)	**0.007**
LAH ^#^	166 (21.3)	52 (36.1)	23 (20)	**<0.001**
QRS duration *	100 (80; 110)	110 (90; 140)	130 (100; 150)	**<0.001**
PQ interval *	170 (150; 200)	180 (150; 220)	190 (162; 217)	0.116
Resting heart rate (bpm) *	71 (63; 81)	70 (61; 78)	60 (60, 70)	**0.047**
**Echo-parameters (prior to intervention):**				
LVEF (%) *	60 (50, 60)	60 (60, 60)	55 (35, 60)	**<0.001**
AVmean Gradient (mmHg) *	44 (36; 55)	44 (37; 52)	40 (30; 51)	0.064
AVA (cm^2^) *	0.7 (0.6; 0.8)	0.7 (0.6; 0.8)	0.7 (0.6; 0.9)	**0.017**
AV Vmax (m/s) *	4.2 (3.8; 4.6)	4.2 (3.8; 4.5)	4.0 (3.5; 4.5)	0.060
**Procedural parameters**				
Valve type				
Edwards SAPIEN 3 THV-Model 9600 TFX ^#^	440(52.4)	83 (54.2)	73 (60.8)	
Edwards Sapien 3 Ultra THV-Model 9750 TFX ^#^	259 (30.8)	36 (23.5)	27 (22.5)	
Edwards SAPIEN XT THV-Model 9300 TFX ^#^	4 (0.5)	2 (1.7)	2 (1.3)	
Portico™ Transcatheter Aortic Valve ^#^	76 (9.0)	17 (11.1)	13 (10.8)	
Evolut™ R system ^#^	23 (2.7)	5 (3.3)	2 (1.7)	
Evolut™ PRO+ system ^#^	5 (0.6)	1 (0.7)	0 (0.0)	
LOTUS Edge™ Aortic Valve System ^#^	31 (3.7)	9 (5.9)	3 (2.5)	
ALLEGRA™ TAVI System TF ^#^	2 (0.2)	0 (0.0)	0 (0.0)	0.467
Aortic valve size (mm) ^#^	26.2 ± 2.2	26.9 ± 2.2	27.2 ± 2.0	**<0.001**
Type of expansion				
Balloon-expandable ^#^	704 (83.7)	121 (79.1)	102 (85.0)	
Self-expanding ^#^	106 (12.6)	23 (15.0)	15 (12.5)	
Mechanically expandable ^#^	31 (3.7)	9 (5.9)	3 (2.5)	0.516
Balloon pre-dilatation ^#^	20 (2.4)	2 (1.3)	3 (2.5)	0.699
Balloon post-dilatation ^#^	86 (10.2)	15 (9.8)	10 (8.3)	0.809
Complications				
Annular rupture ^#^	2 (0.2)	1 (0.7)	0 (0.0)	0.550
Cardiac arrest ^#^	2 (0.2)	1 (0.7)	0 (0.0)	0.550
Aortic dissection ^#^	1 (0.1)	0 (0.0)	0 (0.0)	0.850
Conversion to open surgery ^#^	2 (0.2)	0 (0.0)	1 (0.8)	0.393

AF, Atrial fibrillation; AV, Aortic valve; AVA, Aortic valve area; BMI, Body mass index; CAD, Coronary artery disease; CRP, C-reactive protein; DM, Diabetes mellitus; LAH, Left anterior hemiblock; LBBB, Left bundle branch block; LVEF, Left ventricular ejection fraction; No PM, No pacemaker neither before nor after TAVI; NT-proBNP, N-terminal pro-brain natriuretic peptide; Post-PM, New pacemaker after TAVI; Pre-PM, Pre-existing pacemaker; PAD, Peripheral artery disease; RBBB, Right bundle branch block; THV, Transcatheter heart valve; TF, Transfemoral; Vmax, Maximum velocity; ^#^ *n* (%), ^##^ mean ± SD, * median (IQR). Statistical significant values highlighted in bold letters.

**Table 2:** Clinical and demographic data of the AUTHEARTVISIT cohort.

	No PM *n* = 6089	Post-PM *n* = 776	*p*-Value
**Demographic data**			
Age (years) *	81 (78–85)	82 (78–85)	0.107
Gender (female) ^#^	3400 (55.8)	403 (51.9)	0.043
**Comorbidities**			
Heart failure ^#^	1422 (23.3)	190 (24.4)	0.512
ACS ^#^	122 (2.0)	15 (1.9)	1
Stroke ^#^	113 (1.8)	14 (1.8)	1
Diabetes mellitus ^#^	1206 (19.8)	154 (19.8)	1
Obesity ^#^	347 (5.7)	53 (6.8)	0.236
Hyperlipidemia ^#^	1494 (24.5)	218 (28.0)	0.035
Hyperuricemia ^#^	224 (3.6)	38 (4.9)	0.117
Valvular CMP ^#^	5571 (91.4)	718 (92.5)	0.364
Ischemic CMP ^#^	3213 (52.7)	408 (52.5)	0.951
Artherosclerosis ^#^	506 (8.3)	64 (8.2)	1
Pulmonary diseases ^#^	382 (6.2)	52 (6.7)	0.702
Kidney diseases Yes ^#^	1235 (20.2)	176 (22.6)	0.131
Malignant diseases ^#^	385 (6.32%)	49 (6.31%)	1

ACS, Acute coronary syndrome; CMP, cardiomyopathy; No PM, No pacemaker neither before nor after TAVI; Post-PM, New pacemaker after TAVI; ^#^ *n*; * median (IQR).

**Table 3:** Logistic regression analysis of PM implantation within 30 days after TAVI.

		Univariate			Multivariate		
		OR	CI 95%	*p*-Value	OR	CI 95%	*p*-Value
Age	≥82 years	**1.4**	1.0–2.0	**0.040**	1.1	0.7–1.7	0.441
Sex	Male	1.2	0.9–1.7	0.172			
DM		0.7	0.5–1.1	0.171			
CAD		0.8	0.6–1.2	0.362			
PAD		1.6	0.9–3.0	0.077			
AF		0.9	0.6–1.3	0.817			
Euroscore II	≥5 points	1.1	0.7–1.6	0.465			
Troponin T (µg/L)	≥24 pg/mL	1.2	0.8–1.8	0.201			
NT-proBNP (ng/L)	≥1532 ng/L	0.8	0.5–1.1	0.273			
Creatinine (mg/dL)	≥1.3 mg/dL	1.2	0.9–1.8	0.148			
RBBB		**11.9**	7.1–20.1	**<0.001**	**12.1**	6.8–21.4	**<0.001**
LBBB		0.6	0.3–1.3	0.267			
LAH		**2.0**	1.4–3.0	**<0.001**	**1.6**	1.0–2.5	**0.020**
LVEF	<60%	**1.6**	1.0–2.4	**0.020**	**1.9**	1.1–3.0	**0.007**
Prosthesis size	>26 mm	**1.7**	1.2–2.4	**0.002**	**1.8**	1.2–2.7	**0.002**
Balloon pre-dilatation		0.5	0.1–2.3	0.426			
Balloon post-dilatation		0.9	0.5–1.7	0.916			

AF, Atrial fibrillation; CAD, Coronary artery disease; CI, Confidence interval; DM, Diabetes mellitus; LVEF, Left Ventricular Ejection fraction; LAH, Left anterior hemiblock; LBBB, Left bundle branch Block; NT-proBNP, N-terminal pro-brain natriuretic peptide; OR, Odds ratio; PAD, Peripheral artery disease; RBBB, Right bundle branch block. Statistical significant values highlighted in bold letters.

**Table 4:** Cox regression analysis for 1-year mortality.

		Univariate			Multivariate		
		HR	CI 95%	*p*-Value	HR	CI 95%	*p*-Value
**1-year all-cause mortality**							
PM	No PM	1.0					
	Post-PM	1.1	0.7–1.9	0.455			
	Pre-PM	0.9	0.5–1.7	0.975			
Age	≥82 years	1.1	0.8.1.6	0.319			
Gender	Male	0.9	0.6–1.3	0.775			
DM		1.0	0.7–1.4	0.976			
CAD		1.1	0.8–1.6	0.375			
PAD		0.9	0.7–1.3	0.956			
AF		0.7	0.2–2.0	0.590			
CKD		**2.6**	1.4–4.9	**0.002**	1.4	0.5–3.8	0.511
Euroscore II	≥5 points	**1.7**	1.2–2.4	**0.002**	1.1	0.7–1.8	0.515
Troponin T	≥24 pg/mL	**3.3**	2.1–5.3	**<0.001**	**2.2**	1.3–3.8	**0.002**
NT-proBNP	≥1532 ng/L	**2.8**	1.8–4.4	**<0.001**	**2.2**	1.3–3.8	**0.002**
Creatinine	≥1.3 mg/dL	**1.6**	1.2–2.3	**0.003**			
RBBB		**1.3**	0.7–2.4	0.372			
LBBB		0.8	0.4.1.5	0.546			
LAH		1.1	0.7–1.7	0.403			
LVEF	<60%	**1.7**	1.2–2.4	**<0.001**	1.1	0.7–1.7	0.629

Significant variables of the univariate analysis were included in the multivariate analysis. AF, Atrial fibrillation; CAD, Coronary artery disease; CI, Confidence interval; CKD, Chronic kidney disease; DM, Diabetes mellitus; LVEF, Left ventricular ejection fraction; LAH, Left anterior hemiblock; LBBB, Left bundle branch block; HR, Hazard ratio; NT-proBNP, N-terminal pro-brain natriuretic peptide; PAD, Peripheral artery disease; RBBB, Right bundle branch block. Statistical significant values highlighted in bold letters.

**Table 5:** Cox regression analysis for 5-year mortality.

		Univariate			Multivariate		
		HR	CI 95%	*p*-Value	HR	CI 95%	*p*-Value
**5-year all-cause mortality**							
PM	No PM	1.0					
	Post-PM	1.2	0.9–1.5	0.181	1.2	0.8–1.6	0.220
	Pre-PM	**1.6**	1.2–2.1	**<0.001**	**1.4**	1.0–1.9	**0.030**
Age	≥82 years	**1.3**	1.1–1.6	<**0.001**	**1.3**	1.1–1.7	**0.004**
Sex	Female	1.0	0.8–1.2	0.556			
DM		1.1	0.9–1.3	0.170			
CAD		1.1	0.9–1.3	0.286			
PM		1.1	0.9–1.3	0.083			
AF		**1.5**	1.3–1.9	**<0.001**	**1.3**	1.0–1.7	**0.006**
CKD		**2.2**	1.4–3.2	**<0.001**	**2.0**	1.1–3.8	**0.020**
Euroscore II	≥5 points	**1.5**	1.2–1.8	**<0.001**	1.2	0.9–1.5	0.159
Troponin T	≥24 pg/mL	**2.0**	1.6–2.4	**<0.001**	**1.6**	1.2–2.0	**<0.001**
NTproBNP	≥1532 ng/L	**1.8**	1.5–2.3	**<0.001**	**1.4**	1.1–1.8	**0.006**
Creatinine	≥1.3 mg/dL	**1.5**	1.3–1.9	**<0.001**			
RBBB	Yes	1.3	0.9–1.9	0.071			
LBBB	Yes	1.3	0.9–1.5	0.072			
LAH	Yes	1.2	0.7–1.7	0.096			
LVEF	<60%	**1.5**	1.2–2.8	**<0.001**	1.0	0.8–1.3	0.656

Significant variables of the univariate analysis were included in the multivariate analysis. AF, Atrial fibrillation; CAD, Coronary artery disease; CI, Confidence interval; CKD, Chronic kidney disease; DM, Diabetes mellitus; LVEF, Left ventricular ejection fraction; LAH, Left anterior hemiblock; LBBB, Left bundle branch block; HR, Hazard ratio; NT-proBNP, N-terminal pro-brain natriuretic peptide; PAD, Peripheral artery disease; PM, pacemaker; RBBB, Right bundle branch block. Statistical significant values highlighted in bold letters.

**Table 6:** Overview of key studies on post-TAVI permanent pacemaker implantation (PM) and clinical outcomes.

Study	Design & Population	Study Period	Valve Types	Post-PM Rate	Follow-Up	Mean Age	Main Findings	Limitations
**Studies reporting no significant effect of post-TAVI PM on mortality**								
Present Study (Lamm et al. 2025)	Single-center prospective cohort, *n* = 1114 + validation in AUTHEARTVISIT (*n* ≈ 8000)	2011–2022	84% balloon-expandable, 12% self-expanding, 4%	14%	Median 4.2 y	81 ± 6	Post-PM not associated with increased 1- or 5-year mortality; confirmed in national dataset; Pre-PM significantly associated with worse outcomes	No pacing burden or CT-based depth data; no cause-specific mortality or HF hospitalization analysis
SWEDEHEART [12]	Nationwide registry, *n* = 3420	2008–2018	38% balloon-expandable, Self-expandable (not reported)	14%	Median 2.7 y Up to 10 y	82 ± 7	No significant differences in mortality, HF hospitalization, or endocarditis between Post-PM and No-PM groups	No pacing burden data; older generation devices
Hochstadt et al. [13]	Single-center study, *n* = 1489	2009–2019	Balloon-expandable (not reported) 62% self-expandable	19%	Up to 6 y	80 ± 7	No significant association between Post-PM and long-term mortality, even at high pacing burden; Pre-PM significantly associated with higher mortality	Retrospective design, no CT-based implantation-depth assessment
Chen (2024) PARTNER 2 S3 [18]	Multicenter registry, USA, *n* = 857	2014–2017	Balloon-expandable (SAPIEN 3 only)	12.5%	60 Months	PM 83 ± 5 No PM 81 ± 7	No increased long-term mortality with post-TAVI PM; outcomes mainly driven by comorbidities	Limited sample size, device-specific, retrospective design
Wasim (2025) TAVI-NOR [19]	Single-center *n* = 548	2012–2019	≈70% self-expanding, 18% mechanically expandable	31.5%	84 Months	80 ± 7	No significant difference in 7-year mortality between post-PM and no-PM groups	Small cohort, high SEV share, retrospective
Myat (2021) UK-TAVI [17]	Nationwide registry, *n* = 6815	2007–2015	≈60% balloon-expandable, ≈40% self-expanding	19.2%	84 Months	PM 82 ±7 No PM 81 ±7	No independent association of post-PM with long-term mortality after adjustment	Retrospective design, limited granularity of procedural data
**Studies reporting increased risk associated with post-TAVI PM**								
Swiss TAVI [9]	Nationwide registry, *n* = 13,360	2011–2022	49% balloon-expandable, 47% self-expanding, 2.5% mechanically expandable	20%	Median 4.9 y 120 Months	81 ± 6	Post-PM associated with higher all-cause mortality; absolute survival differences small; Kaplan–Meier curves cross multiple times	No valve-specific subgroup analyses, no pacing-burden data, potential non-proportional hazards
Danish Study [10]	Single-center study, *n* =816	2007–2017	9% balloon-expandable, 83% self-expanding, 8% mechanically expandable	16%	Median 3.5 y	81 ± 7	Post-PM associated with increased long-term mortality, HF hospitalization, and reduced LVEF	High SEV share, limited generalizability, retrospective Design
Auffret (2024) FRANCE-TAVI [20]	Nationwide retrospective registry, *n* = 34,717	2013–2019	≈60% balloon-expandable, ≈40% self-expanding	20.1%	60 Months	PM 83 ± 6 No PM 82 ± 7	Post-PM associated with higher long-term mortality; absolute effect size modest	Retrospective design, no pacing-burden or CT-depth data

CI, Confidence interval; CT, Computed tomography; HR, Hazard Ratio; HF, Heart failure; MI, myocardial infarction; PM, Pacemaker; Post-PM, New pacemaker after TAVI; Pre-PM, Pre-existing pacemaker; RR, Relative risk; SEV, Self-expanding valve.

## Data Availability

Data are available from the corresponding author upon reasonable request.

## Abbreviations

CI	confidence interval
HR	hazard ratio
LBBB	left bundle branch block
LVEF	left ventricular ejection fraction
PM	Pacemaker
Pre-PM	pre-existing pacemaker
Post-PM	new pacemaker within 30 days of TAVI
No PM	no pacemaker neither before nor after TAVI
RBBB	right bundle branch block
TAVI	transcatheter aortic valve implantation
